# Intestinal differentiated mucinous adenocarcinoma of the endometrium with sporadic MSI high status: a case report

**DOI:** 10.1186/s13000-017-0629-0

**Published:** 2017-05-12

**Authors:** Mafalda Trippel, Sara Imboden, Andrea Papadia, Michael D. Mueller, Nando Mertineit, Kirsi Härmä, Alina Nicolae, Erik Vassella, Tilman T. Rau

**Affiliations:** 1 Institute of Pathology, University of Bern, Murtenstr. 31, 3008 Bern, CH Switzerland; 2 Department of Obstetrics and Gynaecology, Inselspital, University of Bern, Bern, Switzerland; 3 Department of Radiology, Inselspital, University of Bern, Bern, Switzerland

**Keywords:** Endometrial cancer, Intestinal differentiation, Mucinous adenocarcinoma, MSI, MLH1 promotor methylation

## Abstract

**Background:**

Intestinal differentiation of primary mucinous adenocarcinoma of the uterine corpus is exceedingly rare in comparison to the approximately 25% rate in endocervical and ovarian mucinous carcinoma. Additionally, little is known about the related genetic and epigenetic alterations, even though large-scale molecular characterisation of the different types of endometrial cancer took place in the TCGA project along the entities defined by the recent WHO classification.

**Case presentation:**

We present a 62-year-old patient harbouring a primary mucinous carcinoma of the uterine corpus with a morphological resemblance to mucinous colorectal adenocarcinoma. The intestinal differentiation was substantiated by CDX2 and CK20 positivity in the absence of PAX8, p16, WT1, p53, ER, PgR, AFP, SALL4 and Glypican3. A high MSI status with MLH1 hypermethylation was revealed by molecular testing.

**Conclusion:**

Intestinal differentiation of mucinous adenocarcinoma of the endometrium is a unique observation. Besides morphology, it obviously can share molecular features of sporadic MSI colorectal cancers. It can be speculated that either CDX2 positive morula formation or intestinal metaplasia of the endometrium as rare conditions might be the origin of carcinogenesis for this type II endometrial cancer. Both conditions were not detectable in this case. Of note, categorising endometrial cancers in genetic subgroups like MSI high cancers alone might lead to the integration of likewise morphologically different tumours like the case presented here with intestinal differentiation. Hence, careful genotype-phenotype correlations are warranted for studies of mucinous adenocarcinoma of the endometrium.

**Electronic supplementary material:**

The online version of this article (doi:10.1186/s13000-017-0629-0) contains supplementary material, which is available to authorized users.

## Background

Intestinal differentiation occurring in endometrial carcinoma is rare. This phenomenon was first described by Berger et al. in 1984 [1] and was confirmed by several case reports [2–5], but it has not undergone implementation into the WHO classification of tumours of the uterine corpus.

According to this morphology-based taxonomy [6], there are eight accepted types of endometrial carcinoma, as follows: endometrioid carcinoma, mucinous carcinoma, serous carcinoma, clear cell carcinoma, mixed cell adenocarcinoma, undifferentiated carcinoma, dedifferentiated carcinoma and neuroendocrine tumours, with a spectrum reaching from low-grade neuroendocrine tumours up to large and small cell high-grade neuroendocrine carcinomas, similar to what has been conceptualised in other organs [7]. The three subtypes in the group of endometrioid carcinomas include cancers with squamous differentiation, as well as villoglandular and secretory carcinomas.

Intestinal differentiation in endometrial cancers has been shown to be exceedingly less common than in ovarian or endocervical cancers [8]. Meanwhile, attempts have been made to learn more from endometrial cancer by investigating the genetic background. The Cancer Genome Atlas Research Network (TCGA) recently postulated a classification according to genomic features into four categories, which are the following: polymerase ε (POLE) ultramutated, microsatellite instability hypermutated, copy-number low and copy-number high [9]. However, the analysis was mostly restricted to classical endometrioid adenocarcinomas and to a lesser extent to serous carcinomas of the endometrium. Other subtypes have been sofar excluded [9].

The microsatellite instable hypermutated tumours are either associated with Lynch syndrome bearing a germline mutation in one of the mismatch repair proteins (MLH1, MSH2, MSH6 and PMS2) or tumours characterised by sporadic hypermethylation of the MLH1 promotor with subsequent blockade of the transcription process and loss of protein expression [6, 10].

However, the correlation between the phenotype and genotype of endometrial cancers is difficult, and there are some very rare subtypes of endometrial carcinoma that are not well represented by these classifications.

We present herein a case of a mucinous adenocarcinoma of the endometrium with morphological and molecular features that resemble an MSI-high adenocarcinoma of the colon.

## Case presentation

### Clinical history

A 62-year-old woman, mother of one child from two pregnancies, presented to our hospital with recent postmenopausal bleeding. She disclosed no intake of hormonal therapy.

The past medical history was unremarkable with an appendectomy in her adolescence, menarche at the age of 12 and menopause starting around the age of 52. Her body mass index was 21.5. The family history presented two cousins with breast cancer at 45 years old and an unknown gynaecological cancer, respectively. In the first degree family members, no relevant disease was present.

**Fig. 1:**
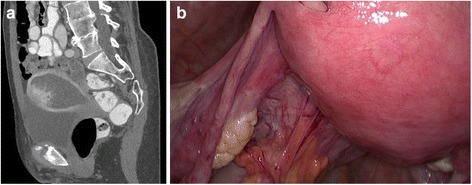
Radiological and intra-operative findings: **a** CT radiography showed a serometra with a polypoid exophytic tumour mass with irregular borders with adjacent organs; **b** the situs during surgical laparoscopy with enlargement of the uterine fundus, inconspicuous serosal surface and ovaries

Initial diagnostics consisted of a preoperative endometrial biopsy and an abdominal CT scan. The latter revealed a mass with clear enlargement of the corpus uteri with serometra, suspicion of infiltrative growth into adjacent organs and no evidence of lymph node or distant metastases (Fig. 1a).

A total laparoscopic hysterectomy, bilateral salpingo-oophorectomy and radical pelvic lymph node dissection was performed. During surgery, extensive exploration of the abdominal organs took place (Fig. 1b).

The post-surgical course was uneventful and the patient was discharged on post-operative day 5. There was no evidence of a gastrointestinal tumour in subsequent gastroscopy, colonoscopy and colposcopy.

Based on the initially completed surgical treatment, no adjuvant treatment was recommended at the interdisciplinary tumour board in accordance with the current guidelines.

Sixteen months after the initial diagnosis, the patient presented herself with back pain and 10 kg of weight loss. A CT scan was performed, which revealed recurrent disease in the pelvis; peritoneal carcinosis was diagnosed. Again, no gastrointestinal primary tumour was evident. The patient refused further diagnostics such as biopsy as well as palliative chemotherapy. She deceased 21 months after initial diagnosis under best supportive care in a palliative care unit.

## Methods

Sections (3 μm thick) were deparaffinised and processed according to routine protocols; details are provided in Table 1. Molecular MSI testing and MLH1 promotor methylation analysis followed standard protocols for Lynch syndrome testing, as published previously [11] with only slight modifications. DNA was extracted using the EZ1 tissue kit and the BioRobot EZ1 workstation (Qiagen, Hilden, Germany). HPV specific PCR was performed according to routine protocols. Six microsatellite markers were assessed as recommended (BAT25, BAT26, D2S123, D17S250, D5S346 and BAT40), and MLH1 methylation status was quantified after bisulfite treatment and corresponding pyrosequencing (bisulfite and pyrosequencing kits, Qiagen, Hilden, Germany).

**Table 1:** Antibodies used for immunohistochemistry

Target	Clone	Company	Dilution	Incubation
CDX2	EPR2764Y	CellMarque	1:400	15 min
CK20	Ks 20.8	CellMarque	1:800	15 min
CK7	OV-TL 12/30	CellMarque	1.800	15 min
Oestrogen	EP1	DAKO	1:50	30 min
MLH1	ES05	Novocastra	1:200	15 min
MSH2	G219-1129	CellMarque	1:500	15 min
MSH6	PU29	Novocastra	1:100	15 min
p16	E6H4	Ventana	1:5	15 min
Pax8	polyclonal	Proteintech	1:200	15 min
PMS2	A16-4	BD Pharmingen	1:100	15 min
Progesterone	16+ SAN27	Novocastra	1:1600	30 min
WT1	6 F-H2	CellMarque	1:100	15 min
AFP	polyclonal	DAKO	1:1600	15 min
SALL4	6E3	Biocare Medical	1:400	15 min
Gypican 3	1G12	CellMarque	1:200	30 min

### Gross features

Macroscopically, the uterus showed a 4.0 × 3.5 × 2.0 cm tumour in the fundus with a glassy mucinous cut surface, with infiltration into the inner half of the myometrium and no extension into the lower uterine segment and cervix. The ovaries and fallopian tubes were unremarkable.

### Microscopic features

**Fig. 2:**
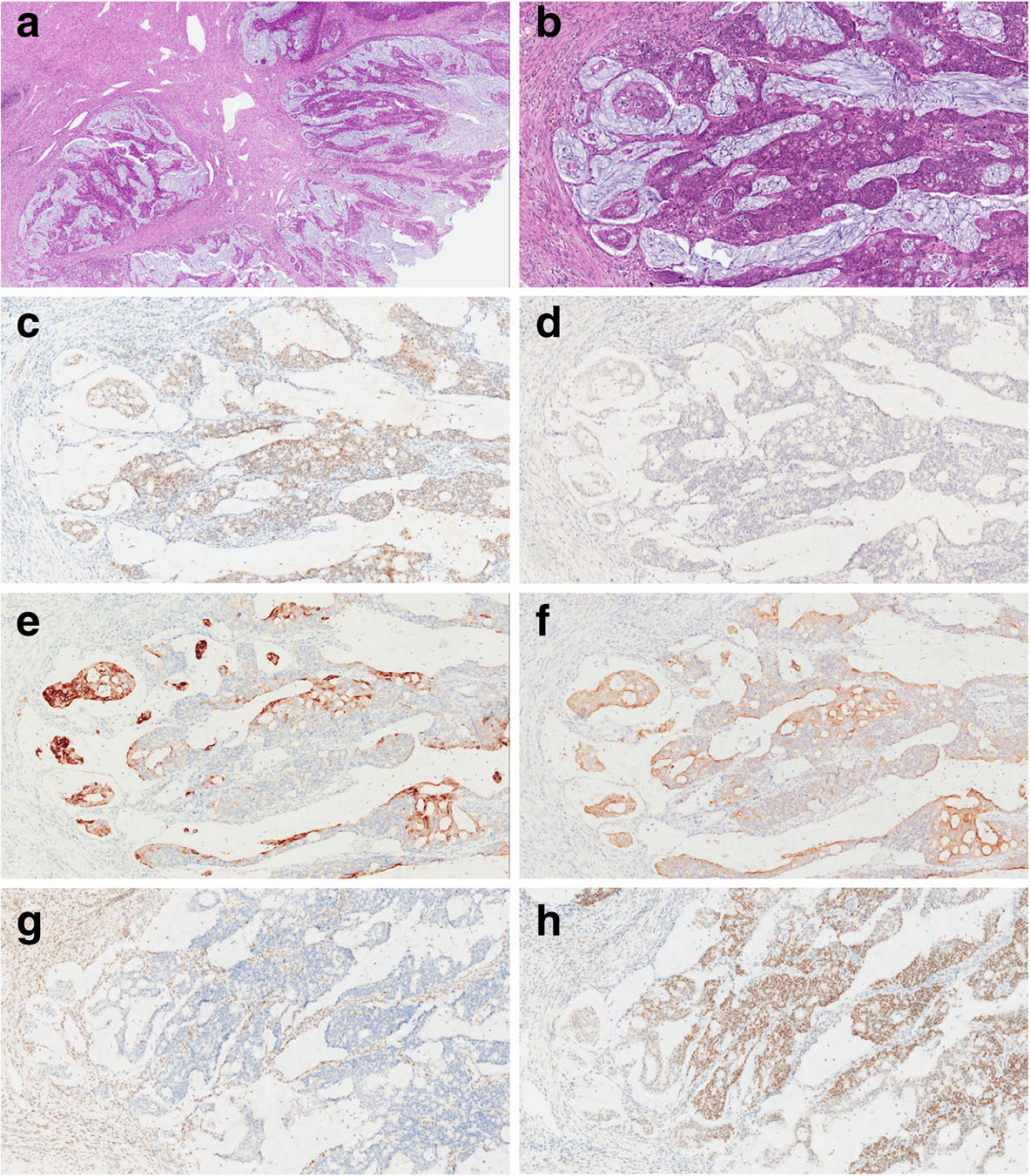
Microscopic findings: H&E sections at low (40×, **a**) and high (200×, **b**) magnification showed mucinous differentiation with major elements of solid and cribriform growth patterns. The immunohistochemical phenotype was characterised by positivity for CDX2 (**c**), and negativity for PAX8 (**d**). Correspondingly, the expression of CK20 (**e**) followed the CDX2 postive areas. However, CK7 (**f**) was also present. MLH1 staining (**g**) showed a protein loss, whereas MSH2 remained positive (**h**). Of note, stromal cells served as positive internal controls for MLH1 and MSH2 (**g**, **h**)

Histologically, the tumour was confined to the uterine cavity. No other neoplasias were present in both ovaries, tubes and the cervix – including no evidence for premalignancies (CIN, AIS). This uterine tumour presented as mucinous adenocarcinoma limited to the inner half of the myometrium with infiltrating borders and lymphatic vessel invasion (Fig. 2 a, b).

The tumour consisted predominantly of highly atypical epithelial cells with a predominantly solid and cribriform growth pattern with abundant extracellular mucin. Applying the regular endometrial grading system, the tumour would have been judged as moderately differentiated (G2), but grading was not finally confirmed due to the intestinal differentiation. Intra-tumoural lymphocytes were slightly increased. Presumably due to tumoural overgrowth we neither found endometrial intraepithelial neoplasi nor intestinal metaplasia nor morula formation as putative precursor lesions.

We observed invasion of small lymphatic vessels, but no evidence of blood vessel invasion nor lymph node or distant metastases. The final staging was defined as: pT1a pN0(0/32) L1 V0 Pn0 Gx R0, FIGO IA.

### Immunohistochemistry

Immunophenotypically (Fig. 2 c-f), the tumour was positive for CDX2 in clusters of up to 60% of tumour cells with partial expression of cytokeratin 7 and cytokeratin 20 in parallel. Cells were negative for PAX8, WT1, synaptophysin, chromogranin and vimentin, as well as for oestrogen and progesterone hormone receptors. Only scattered cells were positive in p16 staining. Yolk sac tumour antigens like AFP, SALL4 and Glypican3 were negative. Interestingly, we observed a complete loss of the mismatch repair proteins MLH1 and PMS2 with retained nuclear expression of MSH2 and MSH6.

### Molecular pathology

According to the molecular analysis, all gene loci of the tumour cells showed a high grade of microsatellite instability and strong hypermethylation of the MLH1 promotor. Additionally, no HPV related DNA was detectable in the tumour.

## Discussion

In the female genital tract, mucinous carcinoma can arise in the vagina, cervix, ovaries and endometrium. Intestinal differentiation seems to be quite common, especially in mucinous adenocarcinomas of the cervix and ovary, with rates up to 25% [8]. This does not account for mucinous carcinoma of the endometrium. The morphological and immunohistochemical evidence for intestinal differentiation in endometrial cancer has only reached the level of single cases [1–3, 5, 8, 12].

The current case of a postmenopausal woman with a hormone receptor negative tumour falls within the spectrum of the clinically relevant group of type II endometrial cancers. In general, carcinogenesis models with the definition of premalignant conditions for type II endometrial cancers (e.g. serous or clear-cell carcinomas) are still being investigated, whereas type I endometrial cancers are believed to evolve stepwise via endometrial intraepithelial neoplasia [6]. Even though this special case of an endometrial type II cancer is extremely rare, it could be correlated to a putative metaplasia-carcinoma sequence. Hence, intestinal metaplasia of the endometrium has been reported as a rare condition and has been regarded as a possible source of malignant transformation [4, 13]. It remains unclear whether milieu factors, inflammation or infections like those known in the upper gastro-intestinal tract [14, 15] might trigger in the endometrium the aberrant expression of CDX2 and the downregulation of hormone receptors and PAX8 as Müllerian markers, but these lesions can be discussed as possible precursors of intestinally differentiated adenocarcinomas of the uterine corpus. Unfortunately, the tumour size and overgrowth masked any putative precursor lesions in the present case.

The differential diagnosis of this case included the already mentioned mucinous adenocarcinomas of other regions of the female genital tract. An extension from a cervical cancer could be excluded as well as spread from the ovaries, as both organ types were completely free of tumours and metaplastic changes. Additionally, the immunohistochemical and molecular profile excludes an HPV association (negativity for p16 and HPV-DNA, Additional file 1: Figure S1) or minour serous carcinoma elements (WT1 negativity and p53 mixed expression). CDX2 positivity was strong and homogenous and not confined to substructures like the reported CDX2 positive morules in conventional endometrioid adenocarcinoma [16]. Nonetheless, this not well-understood phenomenon could be a second putative mechanism of intestinal transdifferentiation in endometrial cancers, but seems to be less likely as no squamoid elements or morules were present.

As further differential diagnosis yolk sac tumour of the endometrium as a very rare condition could be mentioned. These tumours can aberrantly express CDX2 and form a glandular pattern [17]. In rare case reports of extra-gonadal localization in the uterus, they are described in premenopausal patients and might cause a clear cell pattern with resemblance of clear cell adenocarcinoma of the endometrium [18, 19]. As a hallmark they express immunohistochemically AFP, SALL4 and Glypican3 [17]. All these three markers were negative in our tumour (Additional file 1: Figure S1) and no morphological criteria of yolk sac tumour like Schiller-Duval bodies were present. Regarding the age of our patient, the possibility of yolk sac tumour seems to be implausible [20]. Nevertheless, some authors argue for the existence of yolk sac tumour of the endometrium in postmenopausal patients based on AFP expression [17, 21]. However, it has been shown that gastric and colorectal cancers can express AFP as well, which can be used as serological biomarker [22, 23]. Our case report points towards a simply intestinal differentiated adenocarcinoma of the uterus. As the yolk sac serves as embryological ancestor of gastrointestinal organs, there might be an immunohistochemical overlap. Possibly, genetics like the loss of chromosomal region 1p or multimodular molecular characteristics could help, if yolk sac tumour is morphologically considered [24].

As additional possibility, metastasis from the gastrointestinal tract was excluded based on the clinical and imaging studies including gastro- and colonoscopy and control staging during follow-up. Hence, the primary site as endometrial cancer could be confirmed.

The early recidive situation after 16 months and tumour related death of the patient after 21 months emphasises the aggressive course, given the fact that the only initial risk factor of staging was lymphangiosis carcinomatosa.

Previous reports on intestinal differentiation in endometrial cancer revealed heterogeneous tumour elements with interspersed goblet cells [2]. However, the current tumour completely mimicked a mucinous adenocarcinoma of the colon, which are also typically found to be right-sided and hypermutated (MSI high). This molecular status was unequivocally present in our case, with protein loss of MLH1 and PMS2 due to hypermethylation of the MLH1 promotor and functional high microsatellite instability. Thus, the tumour shared not only the morphology, but also the essential genetics of this type of colon cancer.

To the best of our knowledge, this is the first case of a sporadic MSI high endometrial cancer with intestinal differentiation. Whether this special tumour type should be graded according to the endometrial cancer system (moderate grade) or the colon cancer system (low grade) remains unclear.

The recent Cancer Genome Atlas suggests a genetically driven taxonomy of endometrial cancer. This case provides evidence that the category of MSI high hypermutated endometrial cancer might contain so far under represented morphologically diverse cancer subtypes. Of note, only endometrioid and serous carcinomas of the uterine corpus were integrated in the TCGA series. Within these pre-selected cases, little is known about the frequency of intestinal differentiation [9], but this has been reported to be less than 1% elsewhere [8]. Thus, the TCGA platform should be expanded to rare tumour entities like mucinous adenocarcinoma of the endometrium, as in the present case. Additionally, the search for similarities between carcinomas of the uterine corpus, the ovaries and the breast performed within the TCGA project [9] should include other links like gastro-intestinal differentiation and similarities to colorectal cancer.

## Conclusions

In conclusion, this case is an extreme example in which genetics alone could harbour considerable overlap between morphologically completely different cancer subtypes, like this case of an MSI high intestinally differentiated mucinous adenocarcinoma of the endometrium in comparison with a conventional sporadic MSI endometrioid adenocarcinoma. Thus, thorough genotype and phenotype matching for studies in endometrial cancer is essential. As presented above, the WHO classification did not foresee an intestinal differentiated endometrial adenocarcinoma. However, reports of such carcinomas reach back more than 30 years [1] and might be connected to certain premalignant conditions [4, 13]. The link to MSI-genetics and a possible aggressive course warrant its notification as a separate rare variant of mucinous endometrium carcinoma.

## Additional file

Additional file 1: Figure S1. Additional negative immunohistochemical markers, p16 (A), AFP (B), Glypican3 (C) and SALL4 (D). (TIF 6136 kb)

## Abbreviations

MSI: Microsatellite instability
TCGA: The Cancer Genome Atlas
