# Supplementary figures and images for: Intestinal differentiated mucinous adenocarcinoma of the endometrium with sporadic MSI high status: a case report
Source: Diagn Pathol. 2017 May 12;12:39. doi: 10.1186/s13000-017-0629-0 (PMC5427532; doi:10.1186/s13000-017-0629-0)

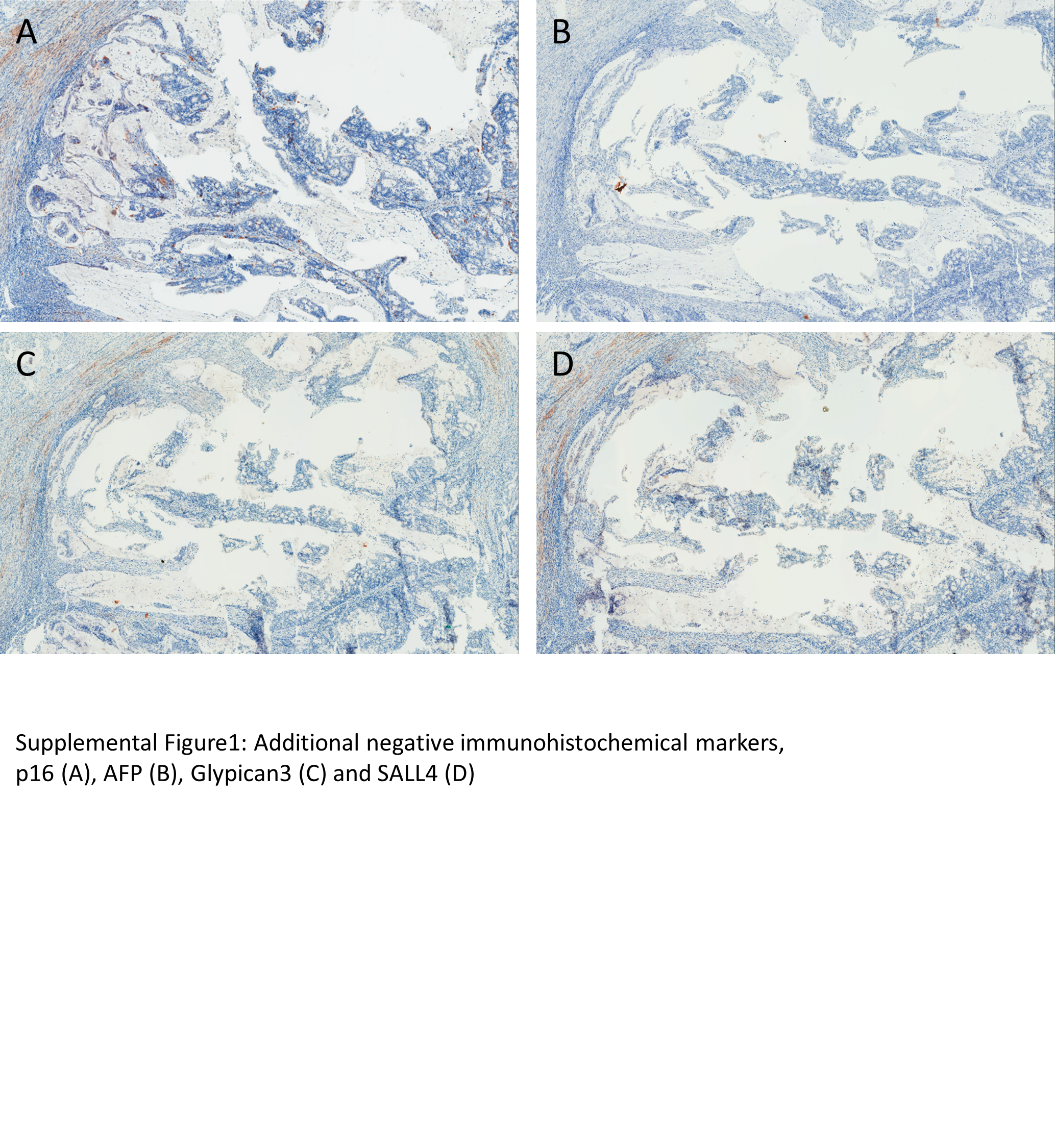

Supplement: Additional file 1: Figure S1. — Additional negative immunohistochemical markers, p16 (A), AFP (B), Glypican3 (C) and SALL4 (D). (TIF 6136 kb) [file 13000_2017_629_MOESM1_ESM.tif]
